# Nurses’ knowledge, attitudes, and practices about rehabilitation of patients after heart valve surgery in Namibia

**DOI:** 10.4102/hsag.v29i0.2396

**Published:** 2024-01-31

**Authors:** Lilian S. Masule, Kristofina Amakali, Wilma E. Wilkinson

**Affiliations:** 1 Department of General Nursing Science, Faculty of Health Sciences and Veterinary Medicine, University of Namibia, Windhoek, Namibia; 2 Department of General Nursing Science, Faculty of Health Sciences and Veterinary Medicine, School of Nursing and Public Health, University of Namibia, Windhoek, Namibia

**Keywords:** knowledge, attitudes, practices, nurses, cardiac rehabilitation

## Abstract

**Background:**

A cardiac rehabilitation programme is a medically supervised intervention to assist patients in recovery after heart surgeries and to prevent potential complications. Nurses should have the knowledge, a positive attitudes, and good practices to improve patient quality of life during the recovery process.

**Aim:**

This study aimed to describe the knowledge, attitudes, and practices of the nurses regarding cardiac rehabilitation for patients after heart valve surgery.

**Setting:**

The study setting was Windhoek Central Hospital, Cardiac Unit, in Windhoek, Namibia.

**Methods:**

A quantitative and descriptive research design was used for convenient non-probability sampling of (*N* = 23) nurses who consented to participate in the study. Data were collected through self-administered questionnaires and analyzed using SPSS Version 26. Descriptive statistics were used and Fischer’s Exact test for associations of variables was performed.

**Results:**

The study results showed a high level of knowledge, good attitudes, and poor to fair practices toward cardiac rehabilitation. However, the respondents demonstrate a lack of knowledge about the indications, and benefits of cardiac rehabilitation, negative attitudes regarding counselling of patients on sexual activities, and poor practice regarding exercises and counseling of patients and caregivers on cardiac rehabilitation. Furthermore, there is a positive relationship between the respondents’ older age, senior rank, and having been trained I cardiac conditions and their knowledge, attitudes and practices regarding some core components of cardiac rehabilitation with P-value < 0.050.

**Conclusion:**

There is a need for a cardiac rehabilitation programme to improve nurses’ attitudes and practices toward cardiac rehabilitation.

**Contribution:**

Understanding the gap in knowledge, attitudes, and practices among the nurses regarding cardiac rehabilitation would guide the Ministry of Health and Social Services (MoHSS) in the implementation of the cardiac rehabilitation for patients after heart valve surgery.

## Introduction

Heart valve repair or replacement surgery is a medical procedure for the treatment of moderate structural damage of the heart valves often following rheumatic fever (Mogotlane et al. 2013).

The surgical treatment of choice for valvular heart disease can be either surgical valve repair or valve replacement when heart valves become floppy and leak. The diseased valve is replaced with either a mechanical (artificial) prosthesis, or biological tissues valve (Cetinkaya et al. 2019; Choudhary, Talwar & Airan 2016).

Although heart valve surgery may improve the patients’ quality of life, it is associated with potential complications, such as thrombo-embolism, bleeding, and endocarditis, to mention a few (Harris, Croe & Cao 2015). Nonetheless, depending on the risks and potential complications involved from heart valve repair and replacement surgeries, patients are expected to regain a normal life and health. Therefore, there is a need for a comprehensive, medically supervised interventions of a long-term cardiac rehabilitation programme, for patients after surgical interventions to improve their physical function, prevent potential complications, and thereby improve patients’ quality of life (Alosaimi, Reyes & Brown 2017; Ahyana, Kritpracha & Thaniwattananon 2013; Dalal, Doherty & Taylor 2015).

The core component of cardiac rehabilitation after heart valve surgery includes medical evaluation or patient assessment, prescribed physical activity and exercise, training, cardiac risk factor modification, diet and nutritional counselling, smoking cessation, and psychological counselling and management, which are implemented in four phases (Dalal et al. 2015; Piepoli et al. 2014).

As members of a multidisciplinary team for cardiac rehabilitation, nurses play a significant role in the implementation of cardiac rehabilitation and should do so based on adequate knowledge, positive attitudes, and efficient practice for the implementation of the core components for the phases of cardiac rehabilitation for them to deliver quality nursing care to patients.

However, Degavi and Bhupali (2015) contended that most nursing staff lacks the knowledge about the rehabilitation of patients after heart valve repair and replacement surgery, suggesting a need for exposing the nurses in the areas of cardiac rehabilitation and this may apply to a Namibian context.

### Nurses’ knowledge regarding cardiac rehabilitation

Studies by Zhu et al. (2020), Kumudah et al. (2020) claim that medical and nursing staff have good knowledge regarding cardiac rehabilitation. In contrast, a study by Salim and Hassoun (2021) indicated that half of the medical staff, including nurses, have poor knowledge about the cardiac rehabilitation programme. Degavi and Bhupali (2015), Choure (2015), De Melo Ghisi et al. (2018) concur that most nursing staff lacked the knowledge about cardiac rehabilitation and were not aware of the importance of its core components for secondary prevention for patients after heart valve repair or replacement surgery.

### Nurses’ attitudes towards cardiac rehabilitation

Salim and Hassoun (2021) and Kumudah et al. (2020) claim that the majority of the medical and paramedical including the nurses had fair attitudes towards cardiac rehabilitation as they believed that referring patients to cardiac rehabilitation programme (CRP) would prevent cardiovascular diseases, reduce hospital readmission of patients, and bring positive impact on patient outcomes. However, Zhu et al. (2020) assert that healthcare providers with lower education level, lower job title, and shorter specialty work experience were associated with a more negative attitude toward the implementation of cardiac rehabilitation.

### Practices of nurses towards cardiac rehabilitation

There is a dearth of studies conducted on the nurse’s practices regarding cardiac rehabilitation; most of the studies were conducted on nurse’s knowledge, attitudes, and perception regarding cardiac rehabilitation. However, a study by Farah, Groot and Pavlova (2021) among medical doctors reported that half of the doctors did not refer patients for cardiac rehabilitation after discharge from the hospital. Non-referring of patients to the cardiac rehabilitation programme by doctors results into nurses being reluctant to offer rehabilitation services, as no patients are being referred by the doctors after heart surgery. Similarly, the findings from this study revealed that nurses were reluctant to counsel the patients to help them cope with recovery after heart valve surgery. In addition, the findings of this study also indicated that nurses were reluctant to involve the caregivers in the cardiac rehabilitation of their family members.

Furthermore, a study conducted by Ahmed et al. (2022) found that nurses had multiple barriers to practice cardiac rehabilitation such as organisational-related barriers, for example, a lack of financial resources, a lack of training about acute phase of cardiac rehabilitation, shortages of staff members, a lack of specific protocols or policies to address phase 1 of cardiac rehabilitation, and a lack of supervision. In addition, a study conducted by Rashidi, Whitehead and Kaistha (2021) found that the nurses perceived the lack of time to engage the patients in their treatment plan impacted their ability to provide quality care to patients.

In conclusion, a lack of the knowledge, negative attitudes, and poor practice regarding cardiac rehabilitation among nurses as reflected upon in the literature review may apply to nurses in Namibia also. Hence, this study aimed to assess the knowledge, attitudes, and practices of nurses at the cardiac unit of Windhoek Central Hospital in Namibia regarding cardiac rehabilitation.

## Aim of the study

The aim of this study was to explore and describe the knowledge, attitudes, and practices of nurses regarding the cardiac rehabilitation of patients after heart valve repair or replacement surgery.

### Specific objectives of the study were to

- Determine the knowledge of nurses regarding the rehabilitation of patients after heart valve repair or replacement surgery
- Find the attitudes of nurses regarding the rehabilitation of patients after heart valve repair or replacement surgery
- Ascertain the practices of nurses regarding the rehabilitation of patients after heart valve repair or replacement surgery

## Research methods and design

### Research design

A quantitative, descriptive and analytical approach was applied to explore and describe the knowledge, attitudes, and practices of nurses regarding rehabilitation of patients after heart valve surgery. Descriptive design was employed to present the accounts of the nurses’ knowledge, attitudes, and practices regarding cardiac rehabilitation. An analytical approach was used to determine the association between the knowledge, attitudes and practices, and the demographic variables of nurses.

### Study setting

The study was conducted at the Windhoek Central Hospital (WCH), Cardiac Unit and Cardiac Clinic. Windhoek Central Hospital is the only National State Referral and teaching hospital in Namibia, with a bed capacity of 855 patients, where open-heart surgeries (OHS) are performed, including treatment of patients who underwent cardiac surgery.

The Cardiac Unit including Cardiac Clinic has a 26 nursing staff members who provide care to patients with cardiac conditions, such as: open heart surgeries, cardiothoracic surgeries, Cardiac catheterization laboratory, services treatment for renal degeneration, and outpatient cardiac clinic services.

### Study population and sampling strategy

The study population was 26 nurses who provide nursing care to patients who had heart valve repair and replacement surgery at the Cardiac Unit and Cardiac Clinic of WCH.

### Sample size and sampling methods

In all, 23 respondents consented to participate in study from a sample of 26. Two nurses refused and one nurse fell under the exclusion criterion of working experience at Cardiac unit and Cardiac Clinic.

### Data collection

#### Research instrument

A self-administered questionnaire comprising 92 items of the Likert Scale was used. The Likert Scale had 4 points (the highest point being 4 and the lowest being 1) of definitely, probably, definitely not, and don’t know to rate the nurses’ knowledge on, about cardiac rehabilitation. The questionnaire consisted of four sections: Section A was about the respondent’s demographic data. Section B consists of 49 knowledge questions about cardiac rehabilitation and was divided into six subsections of: the knowledge regarding cardiac rehabilitation, patient assessment, exercise training, diet and nutritional counselling, smoking cessation and psychological management of the patient.

Section C was about the attitude of nurses towards cardiac rehabilitation and consisted of five subsections about respondents’ attitudes towards cardiac rehabilitation, regarding patients’ assessment and exercise training, Diet or nutritional counseling, and psycho-social management. The Likert scale of 5 points (strongly agree, agree, neutral, disagree, and strongly disagree) was used to rate participants’ attitudes towards cardiac rehabilitation. Strongly agree scores the highest point of 5 and strongly disagree scores the lowest point of 1. There were 13 negative questions and 10 positive questions.

Section D was about nurses’ practices of cardiac rehabilitation and consisted of 20 questions regarding the activities the nurses provide to the patients before and after discharge from the hospital. A 4-Point Likert Scale of never (1), seldom (2), often (3) and always (4) was used to rate participants’ practices towards cardiac rehabilitation.

#### Data collection procedure

Prior to data collection, permission to conduct the research was sought from the Health Research Ethical Committee of the University of Namibia, the Research Ethical Committee of the Ministry of Health and Social Services, and the Office of the Medical Superintendent at WCH. Informed consent was obtained from the participants before the commencement of the data collection, after the purpose, benefits, and data collection process were explained. Participation was voluntary. Anonymity was ensured as the questionnaires were coded and no names were used. The questionnaires took approximately 30–45 min to complete. The completed questionnaires were collected by the senior registered nurse and the researcher, and all were stored by the researcher. The data collection took place between December 2020 and April 2021. The study was conducted over a period of 5 months owing to the coronavirus disease 2019 (COVID-19) pandemic. The pace of contact with the prospective respondents was extended to comply with the measures for prevention of the spread of COVID-19 to the patients and nurses.

#### Data analysis

The data were analysed using Statistical Package for Statistical Social Science (SPSS) Version 26 Norman et al. (1970). In this study, several steps were taken in the data analysis process. Firstly, the raw data were checked for quality, and any missing data were identified and addressed before being entered into the electronic database for analyses. Secondly, the data were then coded, assigned alphanumerical values and analysed. Thirdly, descriptive statistics of frequency and proportional or percentages were performed. Lastly, for the inferential analysis, multiple linear regression was performed to determine the association between the knowledge, attitudes, and practice scores with the demographic characteristics of the nurse respondents.

### Ethical consideration

The study was approved by the University of Namibia Health Research Ethical Review Committee (Ethical Clearance certificate number: SON/486/2019). Permission to conduct the study at WCH, Cardiac unit and Cardiac Clinic was obtained from the Research Ethics Committee of Ministry of Health and Social Services (Ref: 17/3/3), and the Medical Superintendent of WCH (Ethical Clearance number: Ref. 17/3/3) to allow the nurses in the two departments to participate in the study. The ethical principles of respect for persons, beneficence, non-maleficence, and justice as outlined in the Helsinki declaration, adopted in 1964, were applied (Gray & Grove 2021). Code names of the participants were used to maintain anonymity, privacy, and confidentiality.

## Results

### Demographic data

All 23 questionnaire were completed, thus yielding a response rate of 100%. Of the 23 respondents, 21 (91.3%) were female while 2 (8.7%) were male. With regard to age, 8 (34.8%) belonged to the age group of 20–29 years old, while 4 (17.4%) were from the age group of 30–39 years old, whereas 11 (47.8%) were from the age of 40 years and above. Regarding the rank in the nursing profession, 3 (13%) were enrolled nurses, 17 (73.9%) were registered nurses, and 3 (13%) were senior registered nurses. The majority of the participants (*n* = 11, 47.8%) have completed Bachelor’s degree in Nursing Science, 5 (21.7%) have a postgraduate degree in Nursing Science, and 3 (13%) have a Diploma in Nursing Science. In addition, 3 (13%) have a certificate in nursing and midwifery science, while the remaining 1 (4.3%) participant has a master’s degree.

With regard to years of experience, only 9 (39.1%) of the participants have 2 years and below of experience at the Cardiac Unit and Cardiac Clinic, and the remaining 14 (60.9%) have more than 3 years and above of experience at the Cardiac Unit/Clinic. With regard to training related to cardiac conditions, of the 23 participants, a significant number (*n* = 17, 73.9%) have not attended training related to cardiac conditions; only 6 (26%) have attended any training. With regard to the type of training, 3 (13%) of the participants attended cardiothoracic training, while training for cardiopulmonary resuscitation, cardiovascular diseases, and training in International Normalised Ratio (INR) was each attended by only 1 (4.3%) of the respondents as depicted in Table 1.

**TABLE 1:** Demographic characteristics of the participants (*n* = 23).

Variables	Frequency = *n*	%
**Gender**		
Male	2	8.7
Female	21	91.3
**Age of the participants (in years)**		
20–29	8	34.8
30–39	4	17.4
40 and above	11	47.8
**Rank in nursing profession**		
Enrolled Nurse (E/N)	3	13.0
Registered Nurse (R/N)	17	73.9
Senior Registered Nurse (SR/N)	3	13.0
Control Registered Nurse (CRN)	0	0.0
**Educational level**		
Certificate in nursing and midwifery science	3	13.0
Diploma of nursing and midwifery science	3	13.0
Bachelor of Nursing Science clinical (Honours)	11	47.8
PGD in Nursing Science	5	21.7
Master’s Degree	1	4.3
**Years of experience**		
2 years and below 6 months	9	39.1
3 years and above	14	60.9
**Training related to cardiac conditions attended**		
Yes	6	26.1
No	17	73.9
**Type of cardiac training attended**		
Cardiothoracic nursing	3	13.0
Cardiopulmonary resuscitation	1	4.3
Cardiovascular diseases	1	4.3
INR training	1	4.3

*Source:* Masule, L.S., 2024, ‘The development of a programme for nurses to facilitate the rehabilitation of patients who had heart valve repair or replacement surgery in Namibia’, Dissertation, Faculty of Health Sciences and Veterinary Medicine, School of Nursing and Public Health, University of Namibia, Windhoek, Namibia

**TABLE 2:** A summary of the respondents’ knowledge, attitudes, and practices regarding cardiac rehabilitation. A summary of the findings for quantitative.

Nurses’ knowledge regarding cardiac rehabilitation	Frequency = *n*	%
Cardiac rehabilitation: high level of knowledge	21	91.3
Patients’ assessment: high level of knowledge	22	95.7
Exercise training: high level of knowledge	18	78.3
Diet and nutritional counselling: high level of knowledge	21	91.3
Smoking cessation: high level of knowledge	21	91.3
Psychosocial management: high level of knowledge	22	95.7
Overall combined level of knowledge	20	87.0
Average level of knowledge	3	13.0
Low level of knowledge	0	0.0

*Source:* Masule, L.S., 2024, ‘The development of a programme for nurses to facilitate the rehabilitation of patients who had heart valve repair or replacement surgery in Namibia’, Dissertation, Faculty of Health Sciences and Veterinary Medicine, School of Nursing and Public Health, University of Namibia, Windhoek, Namibia

### Knowledge of the nurses regarding cardiac rehabilitation

Table 3 shows that only 5 (21.7%) of the participants were certain, 9 (39.1%) were uncertain, 6 (26.1%) disagreed, and the remaining 3 (13%) respondents did not know whether cardiac rehabilitation is only for patients who had heart valve surgery recently. The responses indicated that only 7 (30.4%) respondents had the knowledge, 6 (26.1%) were almost certain, 7 (30.4%) disagreed and the remaining 3 (13%) did not know that the cardiac rehabilitation programme in phase 1 begins as soon as the patient is discharged from the hospital. The findings indicated that only 5 (21.7%) respondents were certain, 7 (30.4%) were uncertain, 8 (34.8%) disagreed, and the remaining 3 (13%) did not know whether patients who underwent mitral valve repair should receive Warfarin for 1 month.

**TABLE 3:** A summary of the nurses’ level of knowledge regarding cardiac rehabilitation.

Items	Definitely		Probably		Definitely not		Don’t know	
	*N*	%	*n*	%	*n*	%	*n*	%
Cardiac rehabilitation is designed only for those patients who had heart valve surgery.	5	21.7	9	39.1	6	26.1	3	13.0
Phase 1 of the cardiac rehabilitation programme begins as soon as the patient is discharged from the hospital.	7	30.4	6	21.6	7	30.4	3	13.0
Patients with bio-prostheses or mitral repair should receive warfarin for 1 month after cardiac surgery.	5	21.7	7	30.4	8	34.8	3	13.0
Aerobic and resistance exercise training are two types of exercise recommended for cardiac rehabilitation.	6	26.1	6	26.1	4	17.4	7	30.4
Walking, swimming, jogging, stair climbing and lightweight at 6 weeks after heart surgery is safe for patients who underwent cardiac event and heart surgery.	8	34.8	7	30.4	5	21.7	3	13.0

*Source:* Masule, L.S., 2024, ‘The development of a programme for nurses to facilitate the rehabilitation of patients who had heart valve repair or replacement surgery in Namibia’, Dissertation, Faculty of Health Sciences and Veterinary Medicine, School of Nursing and Public Health, University of Namibia, Windhoek, Namibia

The findings indicate that only 6 (26.1%) of the participants were certain, another 6 (21.1%) were uncertain, 4 (17.4%) disagreed, and 7 (30.4%) did not have knowledge about the two types of exercises for cardiac rehabilitation. The findings indicate that only 8 (34.8%) respondents were certain, 7 were uncertain (30.4%) and 5 (21.7%) disagreed, while 3 (13.0%) did not know that walking, swimming, jogging, stair climbing, and lightweight 6 weeks after heart surgery is safe for patients who underwent heart valve surgery.

### Attitudes of nurses regarding cardiac rehabilitation

Table 4 depicts attitudes of respondents towards cardiac rehabilitation. According to the table, majority of the respondents (*n* = 21, 91.3%) had positive attitudes and only 1 (4.3%) had negative attitudes. However, similar to low level of knowledge in some aspects of cardiac rehabilitation, the respondents had negative attitudes in some aspects of cardiac rehabilitation as shown in Table 5. When the respondents were asked to indicate to what extent they believe that rehabilitation should begin as soon as the patient is discharged from the hospital, an average of the respondents (*n* = 12, 52.2%) strongly agreed, 4 (17.4%) agreed, 3 (13%) disagreed, 2 (8.7%) were neutral and 2 (8.7%) strongly disagreed to the statement. Close to two-thirds of the respondents (*n* = 14, 60.9%) strongly agreed, 4 (17.4%) were neutral, 3 (13%) agreed, while the remaining 1 (4.3%) each disagreed and strongly disagreed, respectively, indicating that it was the duty and responsibility of the cardiologists to give the information to patients regarding rehabilitation after cardiac surgery. Only 7 (30.4%) of the participants strongly agreed that the information given to the patients on discharge regarding medications, wound care, infection control, psychological stress is sufficient for their recovery process, 6 (26.1%) disagreed, 5 (21.7%) agreed, 3 (13%) were neutral and the remaining 2 (8.7%) strongly disagreed to the statement.

**TABLE 4:** A summary of the nurses’ overall attitudes towards cardiac rehabilitation.

Item	Frequency = *n*	%
Attitudes towards cardiac rehabilitation have positive attitudes	16	69.6
Patient assessment and exercise training have positive attitudes	22	95.7
Diet and nutritional counselling and psychosocial management have positive attitude towards cardiac rehabilitation.	19	82.6
The overall combination shows that the majority have a positive attitude towards cardiac rehabilitation.	21	91.3

*Source:* Masule, L.S., 2024, ‘The development of a programme for nurses to facilitate the rehabilitation of patients who had heart valve repair or replacement surgery in Namibia’, Dissertation, Faculty of Health Sciences and Veterinary Medicine, School of Nursing and Public Health, University of Namibia, Windhoek, Namibia

**TABLE 5:** Nurses’ attitudes towards cardiac rehabilitation.

Items	Strongly agree		Agree		Neutral		Disagree		Strongly disagree	
	*n*	%	*n*	%	*n*	%	*n*	%	*n*	%
I believe rehabilitation should begin as soon as the patient is discharged from hospital	12	52.2	4	17.4	2	8.7	3	13.0	2	8.7
I believe it is the duty and responsibility of the cardiologists to give information to patients regarding rehabilitation after cardiac surgery.	14	60.9	3	13.0	4	17.4	1	4.3	1	4.3
I think the information given to patients on discharge regarding medications, wound care, infection control, and psychological stress is sufficient for their recovery process.	7	30.4	5	21.7	3	13.0	6	26.1	2	8.7
I think the Warfarin information booklet given to patients after discharge is sufficient in providing information about diet/nutrition counselling services.	9	39.1	5	21.7	2	8.7	5	21.7	2	8.7
I feel uncomfortable to talk to patients and their caregivers about sexual life experiences.	1	4.3	8	34.8	6	26.1	8	34.8	0	0.0
I feel the support and care given to patients by caregivers at home is sufficient for patient recovery and return to normal activities.	5	21.7	3	13.0	9	39.1	5	21.7	1	4.3
I do not think caregivers usually experience any difficulty in support and care of their loved ones who had heart valve surgery.	3	13.0	3	13.0	7	30.4	4	17.4	6	26.1

*Source:* Masule, L.S., 2024, ‘The development of a programme for nurses to facilitate the rehabilitation of patients who had heart valve repair or replacement surgery in Namibia’, Dissertation, Faculty of Health Sciences and Veterinary Medicine, School of Nursing and Public Health, University of Namibia, Windhoek, Namibia

In addition, only 9 (39.1%) respondents strongly agreed and 5 (21.7%) agreed and believed that the Warfarin information booklet given to patients after discharge is sufficient in providing the information about diet/nutrition counselling services. However, 5 (21.7%) disagreed, 2 (8.7%) strongly disagreed and the remaining 2 (8.7%) were neutral to the statement. With regard to sexual life experiences, 8 (34.8%) agreed that they were uncomfortable to talk to patients and their caregivers about sexual life experiences. Similarly, 8 (34.8%) disagreed or were discontented with the statement, while 6 (26.1%) were neutral and only 1 (4.3%) strongly agreed that they feel uncomfortable to talk to patients and caregivers about sexual life. Furthermore, the findings showed that only 5 (21.7%) respondents strongly agreed, a significant 9 (39.1%) were neutral, 3 (13%) agreed, and the remaining 1 (4.3%) strongly disagreed that they feel the support and care given to patients by caregivers at home is sufficient for patient recovery and to return to normal activities. However, 5 (21.7%) participants disagreed to the statement. Lastly, 7 (30.4%) of the respondents, were neutral, while 6 (26.1%) strongly disagreed and 4 (17.4%) disagreed that they do not think caregivers usually experience any difficulty in support and care of their loved ones who had heart valve surgery. Only 3 (13%) respondents strongly agreed and 3 (13%) agreed to the statement.

### Practices of the nurses towards cardiac rehabilitation

The findings in Table 6 indicated that of the respondents, 13 (56.5%) had good practice, 8 (34.8%) had average practice and 1 (4.3%) had poor practice. However, the respondents displayed poor practice in some aspects of cardiac rehabilitation. Table 7 indicates respondents’ response on to what extent the following rehabilitation activities were provided to patients before discharge from the hospital: With regard to early detection of complications, 11 (47.8%) always provide the information, 6 (26.1%) often, 5 (21.7%) seldom and 1 (4.3%) never provides the patients with the information in this regard. In relation to the importance of physical exercise, more than one-third of the respondents (*n* = 9, 39.1%) often talk about physical exercise, 8 (34.8%) always, and the remaining 6 (26.1%) seldom talk to patients about the importance of physical exercise after discharge from the hospital. In addition, in terms of sexual activities counselling, 7 (30.4%) often, 6 (26.1%) seldom, 6 (26.1%) never, and only a mere 4 (17.4%) always provide sexual activities counselling to patients. About providing advice to caregivers regarding support and care of their family members, a considerable proportion of respondents (*n* = 11, 47.8%) always advice caregivers, 7 (30.4%) often, 4 (17.4%) seldom, and the remaining 1 (4.3%) never advice the caregivers on support and care of their family members. In addition, only 11 (47.8%) always, 4 (17.4%) often, 6 (26.1%) seldom, and remaining 2 (8.7%) never worked through the Warfarin information booklet with the patients before discharge from the hospital.

**TABLE 6:** Nurses’ overall practices towards cardiac rehabilitation.

Level of practice	Frequency = *n*	%
Good practice	13	56.5
Average practice	**8**	34.8
Poor practice	**1**	4.3
Missing item	**1**	4.3

**Total**	**23**	**95.7**

*Source:* Masule, L.S., 2024, ‘The development of a programme for nurses to facilitate the rehabilitation of patients who had heart valve repair or replacement surgery in Namibia’, Dissertation, Faculty of Health Sciences and Veterinary Medicine, School of Nursing and Public Health, University of Namibia, Windhoek, Namibia

**TABLE 7:** Nurses practice regarding cardiac rehabilitation.

Items	Never		Seldom		Often		Always	
	*N*	%	*n*	%	*n*	%	*n*	%
Indicate which of the following rehabilitation activities you provide to patients before discharge from the hospital:								
Early detection of complications	1	4.3	5	21.7	6	26.1	11	47.8
Sexual activities counselling	6	26.1	6	26.1	7	30.4	4	17.4
Advice caregivers regarding support and care of their family members.	1	4.3	4	17.4	7	30.4	11	47.8
Preparing a work-up schedule for the activities of the patients regarding cycling, rowing, swimming, bicycling, jogging, and rest.	14	60.9	4	17.4	2	8.7	3	13.0
Counselling of the patients in helping them to cope with heart valve surgery, stress and anxiety	1	4.3	8	34.8	9	39.1	5	21.7
Counselling of the caregivers to help them to cope with stress and anxiety in caring for their family member after heart valve surgery.	3	13.0	6	26.1	11	47.8	3	13.0
Encouraging and counselling the caregivers to get involved in the physical activities of their family member.	2	8.7	6	26.1	10	43.5	5	21.7

*Source:* Masule, L.S., 2024, ‘The development of a programme for nurses to facilitate the rehabilitation of patients who had heart valve repair or replacement surgery in Namibia’, Dissertation, Faculty of Health Sciences and Veterinary Medicine, School of Nursing and Public Health, University of Namibia, Windhoek, Namibia

Furthermore, the participants were asked if they prepared a work schedule for the activities of the patients regarding cycling, rowing, swimming, bicycling, jogging, and rest. Only 3 (13%) always, 2 (8.7%) often, 4 (17.4%) seldom and 14 (60.9%) never prepare a work schedule. Whether they counsel the patients in helping them to cope with heart valve surgery, stress or anxiety, only 5 (21.7%) always, 9 (39.1%) often, 8 (34.8%) seldom, and 1 (4.3%) never counsel the patients. Whether they counsel the caregivers to help them cope with stress and anxiety in caring for their family members after heart valve surgery, 3 (13%) always, 11 (47.8%) often, 6 (26.1%) seldom and 1 (4.3%) never counsel the caregivers. Furthermore, the respondents were asked if they normally encourage caregivers’ involvement in the physical activities of their family members and 10 (43.5%) often encourage and counsel caregivers, 6 (26.1%) seldom, 4 (17.4%) always, and the remaining 2 (8.7%) never encourage caregivers’ involvement in the physical activities of their family members.

Table 2 shows that the overall level of knowledge regarding aspects of cardiac rehabilitation indicates that the majority (95.7%) of the respondents have the knowledge on patient assessment and psychosocial management, respectively, followed by 91.3% who have the knowledge on cardiac rehabilitation, diet and nutritional counselling and smoking cessation, respectively, while the knowledge on exercise training indicated that 78.3% of the respondents had high level of knowledge. In addition, the overall combined level of knowledge was 87%, average 13% and 0% on low level of knowledge.

Table 4 indicates that the nurses overall combined level of attitudes towards cardiac rehabilitation shows that the majority (*n* = 21, 91.3%) had positive attitudes and only 1 (4.3%) had negative attitudes.

Table 8 shows the association between the nurses’ age and attitudes towards cardiac rehabilitation regarding some aspects of the core components of the cardiac rehabilitation. The association between nurses’ age and attitudes was determined to establish whether the respondents were skilled enough and interested to give health information to patients regarding cardiac rehabilitation programme after heart valve surgery. About 72.7% of the respondents of 40 years old and above had positive attitudes towards cardiac rehabilitation. Therefore, a statistically significant association (*p*-value < 0.050) was found between the age group above 40 years old and positive attitude.

**TABLE 8:** Association between nurses’ age and aspects of attitudes towards cardiac rehabilitation.

Item	Nurses Age	Strongly agree		Agree		Neutral		Disagree		Strongly disagree		Fishers Exact Test *p*
		*n*	%	*n*	%	*n*	%	*n*	%	*n*	%	
I think I am skilled enough and interested to give health information to patients regarding cardiac rehabilitation programme after heart surgery.	20–29	2	25.0	0	0.0	5	62.5	1	12.5	0	0.0	0.038*
	30–39	1	25.0	1	25.0	1	25.0	1	25.0	0	0.0	
	40 and above	8	72.7	1	9.1	1	9.1	0	0.0	1	9.1	
I think patients and caregivers should be responsible for their own health behaviour changes, risk reduction, self-management and post-hospitalisation.	20–29	4	50.0	0	0.0	2	25.0	1	12.5	1	12.5	0.015*
	30–39	0	0.0	4	100.0	0	0.0	0	0.0	0	0.0	
	40 and above	5	45.5	4	36.4	0	0.0	2	18.2	0	0.0	
I do not think caregivers usually experience any difficulty in support and care of their lovedones who had heart valve surgery.	20–29	0	0.0	1	12.5	1	12.5	1	12.5	5	62.5	0.025*
	30–39	0	0.0	2	50.0	1	25.0	1	25.0	0	0.0	
	40 and above	3	27.3	0	0.0	5	45.5	2	18.2	1	9.1	

*Source:* Masule, L.S., 2024, ‘The development of a programme for nurses to facilitate the rehabilitation of patients who had heart valve repair or replacement surgery in Namibia’, Dissertation, Faculty of Health Sciences and Veterinary Medicine, School of Nursing and Public Health, University of Namibia, Windhoek, Namibia

Note: The number of valid cases = 23.

* , indicates a significant relationship between variables.

In addition, a negative association between attitude and age regarding the encouragement of health behaviour changes, risk reduction, and self-care management of patients and caregivers for rehabilitation was found across all age categories as indicated by the *p*-value of less than 0.050. The study concluded that all the three groups, above 40 years (81.9%), 20–29 years (50%), and 30–39 years (100%), had negative attitudes towards the encouragement of health behavioural changes, risk reduction, and self-care management of patients and caregivers.

Furthermore, there is a significant association between nurses’ age categories of 20–29 years and positive attitudes towards cardiac rehabilitation as indicated by a *p*-value of less than 0.050. This was evident in nurse respondents who believed that caregivers usually experience difficulty in supporting and caring for their relatives after heart valve surgery.

Table 9, shows that there is association between nurses’ gender (female) and negative attitudes about the commencement of CR and the need for continuous provision of information to patients and caregivers as indicated by the *p*-value of less than 0.050 across all the genders.

**TABLE 9:** Association between nurses’ gender and aspects of attitudes towards cardiac rehabilitation.

Item	Nurses rank	Strongly agree		Agree		Neutral		Disagree		Strongly disagree		Fishers exact test *p*
		*N*	%	*n*	%	*n*	%	*n*	%	*n*	%	
I believe rehabilitation should begin as soon as the patient is discharged from hospital.	Male	0	0.0	0	0.0	0	0.0	2	100	0	0.0	0.020*
	Female	12	57.1	4	19.0	2	9.5	1	4.8	2	9.5	
I think the information given to patients on discharge regarding medications, wound care, infection control and psychological stress is sufficient for their recovery process	Male	0	0.0	0	0.0	1	50.0	0	0.0	1	50.0	0.040*
	Female	7	33.3	5	23.8	2	9.5	6	28.6	1	4.8	

*Source:* Masule, L.S., 2024, ‘The development of a programme for nurses to facilitate the rehabilitation of patients who had heart valve repair or replacement surgery in Namibia’, Dissertation, Faculty of Health Sciences and Veterinary Medicine, School of Nursing and Public Health, University of Namibia, Windhoek, Namibia

Note: The number of valid cases = 23.

* , indicates a significant relationship between variables.

Table 10 shows that there was a significant association found between nurses’ rank and attitude regarding the benefit of cardiac rehabilitation. The findings show that about 70.6% of the respondents have positive attitudes toward the benefit of cardiac rehabilitation.

**TABLE 10:** Association between nurses rank and aspects of attitudes towards cardiac rehabilitation.

Item	Nurses rank	Strongly agree		Agree		Neutral		Disagree		Strongly disagree		Fisher’s exact test *p*
		*n*	%	*n*	%	*n*	%	*n*	%	*n*	%	
I am sceptical about the benefits of cardiac rehabilitation.	Enrolled nurse	1	33.3	1	33.3	1	33.3	0	0.0	0	0.0	0.040*
	Registered nurse	1	5.9	0	0.0	4	23.5	7	41.2	5	29.4	-
	Senior registered nurse	1	33.3	1	33.3	0	0.0	1	33.3	0	0.0	-
I do not think caregivers usually experience any difficulty in support and care of their loved ones who had heart valve surgery.	Enrolled Nurse	1	33.3	1	33.3	1	33.3	0	0.0	0	0.0	0.018*
	Registered nurse	0	0.0	2	11.8	6	35.3	3	17.6	6	35.3	-
	Senior registered nurse	2	66.7	0	0.0	0	0.0	1	33.3	0	0.0	-

*Source:* Masule, L.S., 2024, ‘The development of a programme for nurses to facilitate the rehabilitation of patients who had heart valve repair or replacement surgery in Namibia’, Dissertation, Faculty of Health Sciences and Veterinary Medicine, School of Nursing and Public Health, University of Namibia, Windhoek, Namibia

Note: The number of valid cases = 23.

* , indicates a significant relationship between variables.

In relation to whether caregivers experience any difficulty in support and care of a relative after heart valve surgery, about half (52.9%) of the registered nurse have a positive attitude while senior registered nurses have negative attitude (66.7%) in the realisation that caregivers experience difficulties in supporting and caring of their relative after heart valve surgery.

Table 11 shows an association between training related to cardiac condition and a negative attitude towards the benefit of CR as indicated by the *p*-value less than 0.050.

**TABLE 11:** Association between training and other aspects of attitudes towards cardiac rehabilitation.

Item	Training related to cardiac conditions	Strongly agree		Agree		Neutral		Disagree		Strongly disagree		Fisher’s exact test *p*
		*n*	%	*n*	%	*n*	%	*n*	%	*n*	%	
I do not see cardiac rehabilitation as part of my responsibilities.	Yes	0	0.0	2	33.3	2	33.3	0	0.0	2	33.3	0.054
	No	0	0.0	0	0.0	3	17.6	5	29.4	9	52.9	-
I am sceptical about the benefits of cardiac rehabilitation.	Yes	3	50.0	1	16.7	1	16.7	0	0.0	1	16.7	0.008*
	No	0	0.0	1	5.9	4	23.5	8	47.1	4	23.5	-

*Source:* Masule, L.S., 2024, ‘The development of a programme for nurses to facilitate the rehabilitation of patients who had heart valve repair or replacement surgery in Namibia’, Dissertation, Faculty of Health Sciences and Veterinary Medicine, School of Nursing and Public Health, University of Namibia, Windhoek, Namibia

Note: The number of valid cases = 23.

* , indicates a significant relationship between variables.

### Association regarding practice towards cardiac rehabilitation

Table 12, indicates that there is an association between nurses age of 40 and above and practices regarding rehabilitation as indicated by the *p*-value of 0.041 to that advice caregivers regarding support and care of their family members. This indicates good practices among older respondents.

**TABLE 12:** Association between nurses’ age and practices towards cardiac rehabilitation.

Item	Nurses age	Always		Often		Seldom		Never		Fishers exact test *p*
		*n*	%	*n*	%	*n*	%	*n*	%	
Advise caregivers regarding support and care of their family members.	20–29	5	62.5	2	25.0	1	12.5	0	0.0	0.041*
	30–39	0	0.0	1	25.0	3	75.0	0	0.0	
	40 and above	6	54.5	4	36.4	0	0.0	1	9.1	

*Source:* Masule, L.S., 2024, ‘The development of a programme for nurses to facilitate the rehabilitation of patients who had heart valve repair or replacement surgery in Namibia’, Dissertation, Faculty of Health Sciences and Veterinary Medicine, School of Nursing and Public Health, University of Namibia, Windhoek, Namibia

Note: The number of valid cases = 23.

* , indicates a significant relationship between variables.

## Discussion

The findings of this study revealed that the majority (91.3%) of the participants were female while 8.7% were male (Table 1). This is similar to the findings of the previous studies by Kumudah et al. (2020), Zhu et al. (2020) in which also majority of the respondents were female. The author suggests that higher proportions of female nurses in the nursing profession is related to the fact that females tend to be more comfortable nursing patients and elderly people (Kumudah et al. 2020).

Most of the respondents from this study were 40 years and above and 60.9% of them had work experiences of 3 years and above. This is in contrast with the other studies where most of the nurses were below the age of 31 years (Kumudah et al. 2020; Salim & Hassoun 2021). This study suggests that most of the respondents have experience in providing nursing care, as a result, they are potentially able to facilitate quality interventions of cardiac rehabilitation for cardiac patients after heart valve surgery.

The findings indicated that of all the respondents, only 4.3% had master’s degree and 4.3% had post-graduate diploma in nursing science, while the majority are holders of undergraduate qualifications in nursing science. Limited number of respondents with advanced qualification indicates a lack of advanced knowledge and skill to provide quality care for complex conditions such as cardiac rehabilitation. As a result, the findings indicated poor practices for cardiac rehabilitation among the respondents. In contrast to this study, Zhu et al. (2020)’s study showed that 15.1% of the participants had diploma, 71.7% had bachelor’s degrees, and 13.2% had master’s degrees in nursing science. Contrarily, Kumudah et al. (2020) reported that 60.9% of the respondents had a diploma, 35.9% had post-basic education, and 3.3% had a degree

### Knowledge of the nurses regarding cardiac rehabilitation

The findings concluded that even though the nurses had a high level of knowledge about cardiac rehabilitation, the nurses lacked the knowledge on some aspects of cardiac rehabilitation, namely on the phases of cardiac rehabilitation, indications of cardiac rehabilitation, and the types of exercises that are safe for patients after heart valve surgery. Respondents also lacked the knowledge about the core components of cardiac rehabilitation. In support of this study, Salim and Hassoun (2021) reported that half of the medical staff and paramedical staff including nurses have poor knowledge about cardiac rehabilitation programme. Moreover, a study by De Melo Ghisi et al. (2018) revealed that although cardiologists were aware of the importance of cardiac rehabilitation programme’s core components for secondary prevention, they nevertheless have poor knowledge about cardiac rehabilitation. However, in contrast to this study, other studies concur that despite the absence of a comprehensive cardiac rehabilitation programme in healthcare settings, medical and nursing staff have good knowledge about the components of cardiac rehabilitation (Farah et al. 2021; Kumudah et al. 2020; Zhu et al. 2020).

The findings of this study indicated that only 6 (26.1%) respondents had good knowledge, 5 (21.7%) had poor knowledge, and the remaining 12 (52.2%) were not sure and did not know whether cardiac rehabilitation was designed only for patients who had heart valve surgery. According to the guidelines and previous studies, cardiac rehabilitation is not only designed for patients who underwent heart valve surgery but also for other groups of patients such as patients with chronic heart failure, patients with heart transplants confirmed with a diagnosis of exertional angina, as well as patients with acute coronary syndrome (Ambrosetti et al. 2021; Dalal et al. 2015; Piepoli et al. 2014). These results are consistent with those of a study conducted by Degavi et al. (2015) among staff nurses who found that the nurses had inadequate knowledge regarding cardiac rehabilitation programme.

The findings indicated that only 7 (30.4%) participants had good knowledge, while the majority had poor knowledge about when phase 1 begins after heart valve surgery. This indicates that nurses lack the knowledge of the different phases of a cardiac rehabilitation programme. Different studies have highlighted the phases of cardiac rehabilitation. Phase 1 of cardiac rehabilitation begins while the patient is in the hospital and includes early mobilisation for prevention of complications and patient assessment. Phase 2 starts after discharge from the hospital and includes provision of health information to the patients and caregivers. Phase 3 is about exercise and education programme, and lastly, phase 4 is about long-term maintenance (Alosaimi et al. 2017; Kumudah et al. 2020; McMahon, Ades & Thompson 2017). A lack of knowledge by the nurses on the phases of cardiac rehabilitation in this study might indicate non-existing cardiac rehabilitation programme at the Cardiac Unit as well as nurses’ lack of training regarding cardiac rehabilitation of patients after heart valve surgery. Regarding the Warfarin treatment for mitral repair, it was observed that only 5 (21.7%) of the respondents had good knowledge, 7 (30.4%) had poor knowledge, while the majority *n* = 8 (34.8%) were not sure, and the remaining 3 (13%) did not know whether patients who underwent mitral valve repair should receive Warfarin for 1 month. In support of this study, the American Heart Association (AHA) and American College of Cardiology guidelines recommend that following the mitral valve repair, a patient should receive anticoagulants Warfarin during the first 3 months in order to achieve the INR of 2.5 (Members et al. 2021). In contrast, studies found that oral anticoagulants have been associated with major bleeding during the first 3 months of mitral valve repair (Paparella et al. 2016). However, Watt et al. (2020) affirm that postoperative Warfarin administration was associated with improved long-term survival and an overall decrease in the composite bleeding and thromboembolic incidents. Nonetheless, the patients should receive Warfarin during the first 3 months as recommended by the guidelines of International Heart Association.

The findings indicate that only 6 (26.1%) of the participants had good knowledge, and the majority were not sure and had poor knowledge about the two types of exercises for cardiac rehabilitation. This is ascribed to the unavailability of a standardised cardiac rehabilitation programme at the Cardiac Unit to guide the rehabilitation of patients after heart surgery, which might contribute to the nurses’ lack of knowledge of types of exercises for cardiac rehabilitation. International Heart Association has identified and recommended aerobic and resistance exercises as important for the recovery of patients after heart surgery (Ribeiro et al. 2017; Squires 2018; Völler et al. 2015). Moreover, only 8 (34.8%) respondents had good knowledge, and the majority had poor knowledge about types of aerobic exercises. The nurses should be knowledgeable about the importance of physical exercise after heart valve surgery for the recovery of a patient. Walking, swimming, jogging, climbing stairs, and lightweight are types of aerobic exercises. In addition, aerobic exercise for cardiac rehabilitation are recommended by international guidelines and its benefit include enhancing cardiovascular health by lowering and controlling blood pressure, easing chronic pain, maintaining a healthy weight, boosting mood, and strengthening the immune system (Ambrosetti et al. 2021; Grace et al. 2016; Piepoli et al. 2014; Price et al. 2016).

### Attitudes of the nurses regarding cardiac rehabilitation

The findings on the overall respondents’ combined level of attitudes towards cardiac rehabilitation show that majority 21 (91.3%) had positive attitudes and only 1 (4.3%) had negative attitude (Table 4). In support to this study, an analogous study by Salim and Hassoun (2021) also showed that most of the participants had fair and good attitudes towards the cardiac rehabilitation programme.

Despite of the nurse’s positive attitudes towards cardiac rehabilitation, there were some aspects for which the respondents displayed negative attitudes towards cardiac rehabilitation. Respondents believed that cardiac rehabilitation programme should only begin after the patient is discharged from the hospital. However, Kumudah et al. (2020) pointed that phase 1 of cardiac rehabilitation should begin while the patient is still in the hospital. Phase 1 includes patient evaluation or review by cardiologists, the introduction of medications, a discharge plan or guidelines, and provision of information on self-care for the patient to leave the hospital safely (Simon et al. 2023; Varnfield & Karunanithi 2015).

Cardiac rehabilitation has a multidisciplinary team and nurses are part of the team. Nurses are expected to give information to patients about cardiac rehabilitation after cardiac surgery. In contrast, the findings of this study indicated that the nurses believed that it was the duty and responsibility of cardiologists only to give the information to patients regarding rehabilitation after cardiac surgery. The guidelines from the International Heart Association recommended that the delivery of the core components of cardiac rehabilitation requires expertise from a range of different professionals such as cardiologists, general practitioners, nurse specialists, physiotherapists, dieticians, psychologists, exercise specialists, occupational therapist, and clerical administrator (Ambrosetti et al. 2021; British Heart Association 2012; Piepoli et al. 2014; Squires et al. 2018). Therefore, nurses are part of the multidisciplinary team for the delivery of the core components of cardiac rehabilitation.

Gutenbrunner et al. (2022) stated that nursing plays a crucial role in delivering care to the patients by explaining, demonstrating and practising with the goal to assist patients to regain their independence. Similarly, Dalal et al. (2015) further argued that as members of multidisciplinary teams for cardiac rehabilitation, nurses too should play a role in the delivery of the core components of cardiac rehabilitation. Conversely, Kumudah et al. (2020) study reported that more than 60% of the nurses were not aware of their roles and responsibilities in cardiac rehabilitation programme. Therefore, nurses should possess correct knowledge and display a positive attitude towards implementation of the interventions for cardiac rehabilitation to the patients.

Another shortcoming of nurses’ attitude was about the assessment of patients. Half of the nurse respondents had negative attitudes and believed that the information given to the patients on discharge regarding medications, wound care, infection control, and psychological stress is sufficient for their recovery process. In contrast, Berg et al. (2013) suggested the information given to the patients should be on a continuous basis, while in the hospital, after discharge, and during follow-up visits, because patients tend to forget what they were told during hospitalisation, or on discharge. Similarly, Kumudah et al. (2020) stated that healthcare professionals, such as nurses, cardiologists, and other doctors, play a crucial role in assisting patients by offering pre- and post-operative education and counselling and thus assisting them to have fewer worries and discomfort.

Furthermore, only 34.8% of the respondents had positive attitudes and felt comfortable talking to the patients and their caregivers about sexual life. The majority of the respondents were uncomfortable and unsure about talking to patients about sexual activities because they regard the provision of sexual health information to patients as a taboo. This view is in concurrence with a report by Saunamäki and Engström (2014) who concluded that nurses consider sex as a taboo and as a result, they are inclined to dismiss their responsibility to give sexual information to patients. Similar to this study, Pascual et al. (2021) stated that the attitude of the nurses and the setting made it challenging to talk about the sexuality of the patients. The study further stated that respondents believed that sexuality was important, but they were hesitant to talk about it because of taboos, ignorance, and widespread misconceptions (Pascual et al. 2021). In support of this study, nurses’ negative attitudes towards sexual-related health information were also confirmed by an analogous study by Azar, Kroll and Bradbury-Jones (2022) who found that nurses felt uncomfortable as the subject is culturally regarded as a taboo and a sensitive one.

It was observed from this study that only 6 (26.1%) of the respondents had positive attitudes, and the majority had negative attitudes on the support and care given to patients by the caregivers at home as sufficient for patient recovery and return to normal activities. The negative attitude might be because of a lack of training among the nurses on cardiac rehabilitation services. In support of this study, Halm (2016)’ reported that partners complained that they didn’t have much direction regarding their responsibilities or what to expect after discharge from the hospital. Lastly, this study’s findings reported that only 43.5% of the respondents had positive attitudes and the majority had negative attitudes as the nurses do not think caregivers usually experience any difficulties in support and care of their loved ones who have had heart valve surgery. The nurses need to continue supporting the patients and caregivers by providing the information needed for recovery, to avoid uncertainty about their medication regimen when they are discharged from the hospital.

### Practices of the nurses regarding cardiac rehabilitation

Regarding practice, despite good knowledge, the findings indicated that an average 56.5% of the respondents had a good practice, and a sizeable 34.8% had average practice towards cardiac rehabilitation. The findings indicated that the respondents had poor to average practice on some aspects of cardiac rehabilitation. With regard to providing information on early detection of complications, only 47.8% had knowledge. In support of this study, a study by Hameed and Dawood (2022) revealed that the majority of the nurses in the intensive care unit (ICU) had poor knowledge regarding open heart surgery complications. Other findings were on the provision of information with regard to the types of exercises, which are safe for patients. This study further revealed poor practices on the provision of health information regarding sexual activities. Consequently, the study by Azar et al. (2022) advised inclusion of theoretical and clinical foundations of sexuality and sexual health in the nursing school curricula to enhance potential nurses’ competence about the provision of sexually related health education clients. In addition to these findings, only 47.8% of the participants guided patients and caregivers about the use of Warfarin information booklet. According to Shahmoradi et al. (2022), patients who engage in educational strategies before and after undergoing heart surgery can significantly obtain necessary information, and apply such information. As a result, they adhere to treatment regimen and cope with recovery. In support of this study, a study conducted by Gusdal et al. (2016) affirmed that although all registered nurses in hospitals and primary healthcare nurses agreed that family caregivers should be invited to private supportive counselling sessions, none of them did so on a regular basis.

### Association between the respondents’ demographic data, knowledge, attitude and practices regarding cardiac rehabilitation

The findings of this study revealed no relationship between the respondents’ knowledge and demographic characteristics. Thus, unlike Kumudah et al. (2020) who proved otherwise, the findings of this study concur with reports of the analogous studies by Degavi and Bhupali (2015) as well as by Salim and Hassoun (2021) both who found no association between demographic characteristics and nurses knowledge level about cardiac rehabilitation. However, this study indicated significant association between respondents’ attitudes and age, and their professional ranks. Older respondents and those with senior ranks displayed positive attitudes compared with the younger and junior respondents. This is in contrast with the findings of an analogous study by Kumudah et al. (2020) that found no association between nurses demographic characteristics and their attitude towards cardiac rehabilitation.

Furthermore, the findings revealed a significant association between respondents’ demographic characteristics and their practices (*p* = 0.050). The respondents with the age group of 20–29 years and 40 years and above demonstrated a good practice in advising the caregivers regarding support and care of their family members. Subsequently, a rehabilitation programme was developed for nurses to support patients and their caregivers in support of patients’ recovery after heart valve repair and replacement surgery.

## Conclusions

A lack of knowledge about some aspects of cardiac rehabilitation, negative attitude towards some aspects of cardiac rehabilitation, and average-to-poor practice regarding cardiac rehabilitation among the nurse respondents of this study indicated that nurses at the cardiac unit and cardiac clinic are not able to provide comprehensive care to patients after heart valve repair and replacement surgery, for patients to cope with outcomes of surgery. Neither are they able to provide relevant support to the caregivers of patients who had heart valve repair and replacement surgery, for the latter to cope with the demand to care at home. Adverse findings indicate the need for educational information to empower nurses with essential knowledge and skills about cardiac rehabilitation. As a result, a rehabilitation programme was developed for nurses to support patients who had heart valve repair and replacement surgery and their caregivers in support of patients’ recovery after heart valve surgery.

### Limitations

This study is limited to the nurses providing care at Cardiac Unit and Cardiac Clinic at WCH. The study was conducted on a small sample size involving only the State or public nurses providing care at Cardiac Clinic and Cardiac Clinic, therefore, the findings cannot be generalised to other regional hospitals and private hospitals.
